# Modified bi‐weekly cetuximab‐cisplatin and 5‐FU/leucovorin based regimen for effective treatment of recurrent/metastatic head and neck squamous cell carcinoma to reduce chemotherapy exposure of patients

**DOI:** 10.1002/cnr2.1479

**Published:** 2021-06-28

**Authors:** Amichay Meirovitz, Shani Bergerson, Nir Hirshoren, Jeffrey M. Weinberger, Evgeniya Bersudski, Sharon Daniel, Kim Sheva, Cesar A. Perez

**Affiliations:** ^1^ Department of Oncology, Hadassah Medical Center and Faculty of Medicine Hebrew University of Jerusalem Jerusalem Israel; ^2^ Department of Otolaryngology, Hadassah Medical Center and Faculty of Medicine Hebrew University of Jerusalem Jerusalem Israel; ^3^ Department of Pediatrics and Public Health, Faculty of Health Sciences Ben‐Gurion University of the Negev Be'er Sheva Israel; ^4^ Clalit Health Services, Southern Branch Be'er Sheva Israel; ^5^ Division of Medical Oncology Sylvester Comprehensive Cancer Center – University of Miami Miller School of Medicine Miami Florida USA

**Keywords:** cetuximab, cisplatin, fluorouracil, head and neck squamous cell carcinoma, patient chemotherapy exposure

## Abstract

**Background:**

The standard chemotherapy treatment protocol for patients with recurrent/metastatic head and neck squamous cell carcinoma (HNSCC) requires as long as 56 days of hospitalization over six months. Where the 5‐Fluorouracil (5‐FU) pump is available, most treatment will be on outpatient bases, however patients will still be under chemotherapy treatment for a comparable period of time (around 50 days).

**Aim:**

A modified protocol was assessed to decrease hospitalization and/or chemotherapy treatment time without sacrificing outcomes, to potentially increase patient quality of life.

**Methods and results:**

A retrospective analysis (2005–2018) of recurrent/metastatic HNSCC patients with a modified treatment protocol was performed. Treatment consisted of cisplatin, cetuximab, 5‐fluorouracil bolus and leucovorin administered on day 1 of a 2‐week cycle, and a continuous infusion of 5‐fluorouracil on days 1–2 of the cycle. Outcomes were measured by progression‐free survival, overall survival, and patient hospitalization time. Analysis was done using the Kaplan–Meier survival function curve.

The study cohort consisted of 27 patients. The modified treatment protocol resulted in a median progression‐free survival of nine months and median overall survival of 14 months, while hospitalization time was reduced by almost 80% in the first six months of treatment.

**Conclusions:**

Modification of the cisplatin, cetuximab, 5‐FU and leucovorin protocol to a bi‐weekly regimen utilizing alternative drug delivery methods, significantly reduced patient hospitalization from 56 days to 12 days in the first 6 months of treatment. This was achieved without compromising treatment outcome, while significantly reducing the days patients were exposed to chemotherapy, and thus potentially improving quality of life.

## 1 INTRODUCTION

Over 90% of head and neck tumors are squamous cell carcinomas (SSC), accounting for approximately 3% of male cancer worldwide.^1^, ^2^ Up to 60% of recurrences are locoregional and are the leading cause of death, while metastatic recurrences are less frequent and account for up to 30% of cases.^3^, ^4^ The median overall survival (OS) of these cancer patients is approximately 6 months without chemotherapy.^5^, ^6^, ^7^

In the seminal study described by Vermorken et al.^8^ patients with recurrent or metastatic head and neck squamous cell carcinoma (HNSCC) were randomized into one of two treatment groups: The first group received either cisplatin (100 mg/m^2^ of body surface area on day 1) or carboplatin (at an area under the curve of 5 mg/ml/min as a 1‐hour intravenous infusion on day 1) plus fluorouracil (1000 mg/m^2^/day for 4 days) every three weeks for a maximum of six cycles. The second treatment group received the same chemotherapy plus cetuximab (initially at 400 mg/day as a 2‐hour intravenous infusion, then 250 mg/m^2^ as a 1‐hour intravenous infusion per week) for a maximum of six cycles. Patients in this group with stable disease continued to receive cetuximab either until disease progression or until unacceptable toxic effects occurred. The addition of cetuximab to the cisplatin‐fluorouracil foundation treatment increased OS from 7.4 months to 10.1 months when given as a first‐line treatment.^8^ This treatment protocol subsequently became the standard of care for first‐line treatment of HNSCC patients. Even though in the past year pembrolizumab‐based treatment has become the recommended first‐line treatment in majority of relapsed metastatic HNSCC cases, approximately 20% of the patient population who has a combined positive score (CPS) below 1 (low PD‐L1 expression levels), are still entitled to chemotherapy‐based treatment with immunotherapy or to cetuximab‐based therapy as the first‐line treatment. In this population there is no advantage of the immunotherapy over the cetuximab in cancer control. Moreover, as a second‐line treatment after failure of first‐line immunotherapy, a chemotherapy‐cetuximab combination remains the preferred regimen. In the region of Israel where this study took place, it is standard of care to provide 5‐fluorouracil (5‐FU) pumps for outpatient use for a maximum of two days, where longer treatment durations require hospitalization. The expected duration of hospitalization for patients treated with this cetuximab‐based protocol is 48 days over six months. In certain countries however, due to hospital regulations, fluorouracil is given from days 2 to 5 of the cycle thereby extending this hospitalization to 56 days in 6 months. Such a prolonged hospitalization is a concern, especially regarding patient quality of life, and risk of COVID‐19.

In this study, the HNSCC treatment protocol was modified to reduce patient hospitalization duration. Briefly, this modified protocol consisted of administering cisplatin and cetuximab on day 1 of a 2‐week cycle, with a 5‐FU bolus plus leucovorin also given on day 1 of the cycle, and a continuous infusion of 5‐FU on days 1 and 2. The rationale for this modification was derived from experience with cetuximab and fluorouracil in other indications, such as metastatic colorectal carcinoma, or with other combinations, such as docetaxel, in metastatic HNSCC.^9^ In colorectal cancer, cetuximab has shown similar response rates when given in a bi‐weekly or weekly regimen.^10^, ^11^ The modified protocol of this study altered the standard 5 days of 5‐FU continuous infusion to reflect that of the de Gramont protocol, which was effective in colorectal cancer treatment.^12^, ^13^, ^14^

The aim of this study was to successfully utilize a modified, alternative treatment protocol for recurrent and metastatic HNSCC to substantially decrease patient hospitalization duration and subsequently improve patient quality of life.

## 2 MATERIALS AND METHODS

### 2.1 Study population

The study was reviewed and approved by the institutional ethics committee of the Hebrew University‐Hadassah Medical Center, Jerusalem, Israel.

Consecutive medical records of all patients with recurrent or metastatic HNSCC treated at Hadassah Ein Kerem Medical Center (Jerusalem, Israel) between 2005 and 2018, were reviewed. Patients were included in the analysis if they had histologically‐ or cytologically‐confirmed recurrent or metastatic HNSCC, were older than 18 years of age when diagnosed, and were treated according to the modified treatment protocol.

Data collected included date of birth, date of diagnosis of recurrent or metastatic HNSCC, primary tumor site, cancer stage, date of last radiation therapy session, first and last date of treatment with the modified protocol, the start date of any subsequent treatment, and the date of death or last follow up (selected data shown in Supplementary Table 1).

### 2.2 Treatment regimens

The modified treatment protocol comprised administration of 40 mg/m^2^ cisplatin, 500 mg/m^2^ cetuximab, 400 mg/m^2^ 5‐FU bolus and 200 mg/m^2^ leucovorin on day 1 of a 2‐week cycle and a continuous infusion (via an infuser) of 1200 mg/m^2^ 5‐FU on days 1–2 of the cycle. Patients were treated according to the protocol until disease progression or death. Specific drug administration was immediately ceased in patients experiencing any adverse drug reactions, where remaining drug treatment was continued. It was decided to infuse 5‐FU as opposed to administering oral capecitabine because head and neck cancer patients often suffer from dysphagia. A notable change in the modified protocol of this study is the dose intensity and length of cisplatin chemotherapy as compared to the standard ‘Vermorken’ protocol. In the standard protocol 100 mg/m^2^ cisplatin is administered every 21 days for 6 cycles translating into a dose of 200 mg/m^2^ per 4 weeks, as opposed to 80 mg/m^2^ per 4 weeks in the modified protocol. In the modified protocol however, cisplatin is administered for as many cycles as tolerated rather than being limited to only 6 cycles.

### 2.3 Patient monitoring

Patients were monitored every 2.5 to 4 months by computed tomography (CT) or fluorodeoxyglucose positron emission tomography (FDG PET) imaging. Clinical assessments were performed in between radiological assessments. After disease progression, follow ups were performed based on the patient's individual clinical status.

### 2.4 Measurable outcomes

The primary endpoint of the study was OS, defined as the period between starting the modified protocol and death. The secondary endpoint was progression‐free survival (PFS), defined as the period from the first treatment of the modified protocol until proven disease progression.

### 2.5 Statistical analysis

Statistical analysis was performed using SAS software (Cary, NC). Categorical variables were summarized by absolute and relative frequency and continuous variables were summarized by arithmetic mean, standard deviation, median, minimum and maximum values. OS and PFS were analyzed using the Kaplan–Meier survival function curve.

## 3 RESULTS

Of the 238 patients recorded with recurrent or metastatic HNSCC, 27 (70.4% males) were identified as having been treated utilizing the modified protocol and were included in the study. Majority of the study cohort suffered from local regional recurrences of HNSCC (77.8%). The primary cancer sites are summarized in Table 1. The most common site of primary tumors was the oral cavity (63.0%).

**TABLE 1:** Patient demographics and clinical parameters

Parameter		Study population (n = 27)	
Age at diagnosis (years)		61.0	(29–85)
Gender			
Male		19	(70.4%)
Female		8	(29.6%)
Primary tumor location			
Oral cavity		17	(63.0%)
Miscellaneous^a^		5	(18.5%)
Oropharynx		3	(11.1%)
Larynx		2	(7.4%)
Cancer grade			
T0N0M1		5	(18.5%)
T1N2aM0 ECE		1	(3.7%)
T1N2bM0		1	(3.7%)
T2N0M0		2	(7.4%)
T2N1M0		1	(3.7%)
T2N2bM0		1	(3.7%)
T2N2cM0		2	(7.4%)
T3N0M0		2	(7.4%)
T3N1M0		2	(7.4%)
T3N2bM0		1	(3.7%)
T3N2bM1		1	(3.7%)
T4N0M0		3	(11.1%)
T4N1M0		1	(3.7%)
T4N2cM0		1	(3.7%)
T4N2bM0		3	(11.1%)
Prior surgical excision		15	(55.5%)
Prior XRT			
Ave dose post‐surgery		64.1 Gy	
Ave dose w/o surgery		68.7 Gy	
Primary chemo‐radiotherapy		19	(70.4%)
Cisplatin		17	(89.5%)
Cetuximab		2	(10.5%)
Regional recurrence			
Local		21	(77.8%)
Metastatic		6	(22.2%)

*Note*: Categorical variables are shown as n (%), continuous variables are shown as median (range).

^a^ Miscellaneous: 3 patients with nasal sinuses SCC and 2 with inner ear SCC.

Median treatment duration was 6 months. Kaplan–Meier survival analysis showed that median PFS was 9 months with a range of 5–18 months (Supplementary Figure 1). At the time of data cut‐off, four patients had not succumbed to the disease. The Disease Control Rate (DCR) (stable disease plus complete and partial response) was 67% in our cohort. Median OS was 14 months with a range of 7–23 months (Supplementary Figure 2).

Of the 27 patients treated according to the modified protocol, one patient discontinued treatment due to a moderate allergic reaction that was medically managed. An additional patient suffered an anaphylactic reaction to one of the drugs and refused to continue with treatment. Another patient suffered from necrotizing fasciitis of the neck while receiving treatment. This side effect can be attributed to the cancer, the radiation or the treatment received.

Due to the fact that only three patients suffered from grade 3/4 adverse events for which the treatment regimen had to be stopped, no adverse event profiles were included in the study results. The reason that a higher rate of severe adverse events was not seen is most likely related to the fact that the dose intensity of the highly toxic cisplatin was lower in our treatment regimen as compared to the standard regimen.

Utilization of the modified protocol for treatment of HNSCC required only 12 days of patient hospitalization over the first six months.

## 4 DISCUSSION AND CONCLUSION

This study retrospectively assessed a modified cisplatin/cetuximab/5‐FU/leucovorin protocol as a substitute for the standard ‘Vermorken’ protocol.^8^ This modified protocol aimed to improve the quality of life of patients with HNSCC, by significantly reducing patient hospitalization time. The retrospective analysis revealed a median OS of 14 months as compared to the median OS of 10.1 months reported with the standard protocol.

The main achievement of the modified treatment protocol was the reduced length of patient hospitalization. An almost 80% reduction in hospitalization time or outpatient days under chemo treatment was seen as compared to the standard protocol, this might contribute to the improvement of patient quality of life as well as reduced risk of infection. The shorter time of patients having to physically be under chemotherapy treatment can translate into reduced cost of treatment. As opposed to other chemotherapy treatment regimens, no patient hair loss occurred using the modified protocol (except for an allergic reaction in one patient) and no other grade 3–4 toxicities were reported.

The modifications to the standard cetuximab treatment protocol were based on studies that described the use of bi‐weekly cetuximab plus chemotherapy as resulting in relatively comparative or improved effectiveness for different indications. Comparison of bi‐weekly administration of 500 mg/m^2^ cetuximab to the standard weekly cetuximab regimen (initially 400 mg/m^2^ followed by weekly 250 mg/m^2^ dosages) in patients with metastatic colorectal cancer showed that both regimens exhibited comparable pharmacokinetics, exposure and active serum cetuximab concentrations. Additionally, bi‐weekly administration of cetuximab was not associated with higher rates of Grade 3 or 4 adverse events, compared to the standard protocol.^11^

In a Central European Co‐operative Oncology Group (CECOG) phase II study, which investigated first‐line chemotherapy (FOLFOX4) with either standard weekly cetuximab or bi‐weekly cetuximab administration in KRAS wild‐type metastatic colorectal cancer, the objective response rates, PFS, OS and disease control rates were all comparable across both treatment groups. The frequencies of grade 3 and 4 adverse events in both groups were similar.^10^

Posch et al. showed that bi‐weekly administration of 500 mg/m^2^ cetuximab and 50 mg/m^2^ docetaxel is an effective regimen and is well tolerated as a first‐line treatment in recurrent or metastatic HNSCC patients who do not qualify for platinum doublet treatment. Although the study showed lower response and disease control rates as compared to taxane monotherapy, this was attributed to previous chemotherapy treatments that were not included in the monotherapy trials.^9^

The modified treatment protocol of this study also considered studies that compared the de Gramont (LV5‐FU2) protocol with a modified version in advanced colorectal cancer patients. The de Gramont treatment regimen combines a 2‐hour infusion of 200 mg/m^2^ leucovorin with a bolus of 400 mg/m^2^ 5‐FU, followed by continuous infusion of 500 mg/m^2^ 5‐FU, on days 1 + 2 and days 14 + 15 every 4 weeks.^15^ Several trials have assessed the safety and efficacy of this regimen and its modifications. Later, de Gramont et al. reported that a bi‐monthly regimen of leucovorin and 5‐FU was more effective and less toxic in colorectal cancer patients than the monthly regimen of continuous 5‐FU and leucovorin over 5 days with the same drugs.^13^

Cheeseman et al. compared the standard de Gramont regimen to a modified regimen in colorectal cancer patients, where 350 mg d,l‐leucovorin or 175 mg l‐leucovorin was administered as a 2‐hour infusion followed by a bolus of 400 mg/m^2^ 5‐FU, then an ambulatory 46‐hour infusion of 2800 mg/m^2^ 5‐FU, every 14 days. The results showed that the modified regimen was at least comparable to the standard regimen, with the added benefit of requiring only one visit to the hospital every 2 weeks. Combination treatment of this modified de Gramont regimen with 85 mg/m^2^ oxaloplatin showed that response rates were consistent with those obtained with the FOLFOX4 and FOLFOX6 regimens. Both versions of the modified de Gramont regimens were well tolerated.^12^

Limitations of this study include the fact that this was a non‐randomized, one‐armed study, with a relatively small study cohort. Moreover, the clinical diversity of the patients participating in this study was broader than the reference study used for comparison.

Reduction of hospital or outpatient chemotherapy exposure is an aim to achieve across all treatment modalities. This study has shown that modification of the standard cisplatin, cetuximab, 5‐FU HNSCC treatment protocol to a bi‐weekly regimen using alternative drug delivery methods, significantly reduces patient chemotherapy time exposure from approximately 56 days to 12 days in the first 6 months of treatment, without compromising treatment outcome or desired results. Decreasing the time that a patient spends under chemotherapy may not only improve quality of life and reduce total healthcare costs but may improve patient compliance. A prospective clinical trial is warranted to confirm these findings.

## CONFLICT OF INTEREST

The authors have stated explicitly that there are no conflicts of interest in connection with this article.

## ETHICAL STATEMENT

The study was reviewed and approved by the institutional ethics committee of the Hebrew University‐Hadassah Medical Center, Jerusalem, Israel. Informed consent was obtained from all patients whose data was used in the study.

## AUTHOR CONTRIBUTIONS

**Amichay Meirovitz:** Conceptualization; data curation; formal analysis; investigation; methodology; project administration; supervision; writing‐original draft; writing‐review & editing. **Shani Bergerson:** Data curation; formal analysis; methodology; validation; writing‐original draft. **Nir Hirshoren:** Data curation; investigation; writing‐original draft. **Jeffrey Weinberger:** Resources; writing‐original draft; writing‐review & editing. **Evgeniya Bersudski:** Conceptualization; data curation; writing‐original draft. **Sharon Daniel:** Data curation; validation. **Kim Sheva:** Data curation; writing‐original draft; writing‐review & editing. **Cesar Perez:** Data curation; formal analysis; writing‐review & editing.

## Supporting information


**Supplementary Figure 1** Kaplan–Meier analysis of progression‐free survival.

**Supplementary Figure 2** Kaplan–Meier analysis of overall survival.

**Supplementary Table 1** Patient specific details

## Data Availability

The data that support the findings of this study are available on request from the corresponding author. The data are not publicly available due to privacy or ethical restrictions.
