# Supplementary figures and images for: Modified bi‐weekly cetuximab‐cisplatin and 5‐FU/leucovorin based regimen for effective treatment of recurrent/metastatic head and neck squamous cell carcinoma to reduce chemotherapy exposure of patients
Source: Cancer Rep (Hoboken). 2021 Jun 28;5(3):e1479. doi: 10.1002/cnr2.1479 (PMC8955066; doi:10.1002/cnr2.1479)

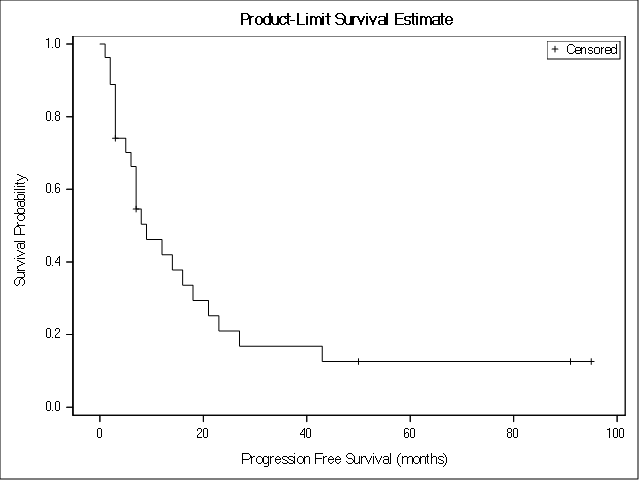


**Supplementary Figure 1. Kaplan-Meier analysis of progression-free survival.**

Supplement: Supplementary file 1 — Supplementary Figure 1 Kaplan–Meier analysis of progression‐free survival. [file CNR2-5-e1479-s003.docx]

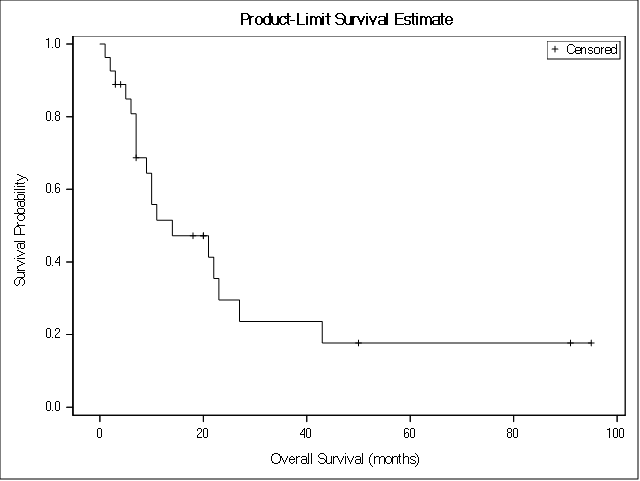


**Supplementary Figure 2. Kaplan-Meier analysis of overall survival.**

Supplement: Supplementary file 2 — Supplementary Figure 2 Kaplan–Meier analysis of overall survival. [file CNR2-5-e1479-s002.docx]
