# Supplementary material for: Modified bi‐weekly cetuximab‐cisplatin and 5‐FU/leucovorin based regimen for effective treatment of recurrent/metastatic head and neck squamous cell carcinoma to reduce chemotherapy exposure of patients
Source: Cancer Rep (Hoboken). 2021 Jun 28;5(3):e1479. doi: 10.1002/cnr2.1479 (PMC8955066; doi:10.1002/cnr2.1479)
Supplement: Supplementary file 3 — Supplementary Table 1 Patient specific details [file CNR2-5-e1479-s001.docx]

**Supplementary Table 1. Patient specific details**

| **Patient** | **EXTREME Regimen Duration** | **ECOG PS** | **Tumor Location** | **Cancer Grade**  **(AJCC-7 staging)** | **Surgery (1-Y; 0-N)** | **XRT Dose** | **Chemotherapy (0-No; 1-Cis, 2-Cet)** |
| --- | --- | --- | --- | --- | --- | --- | --- |
| 1 | 33 weeks | 1 | Tongue | T3N1M0 | 1 | 61,6 Gy | 1 |
| 2 | 27 weeks | 2 | Nasal septum | T3N0M0 | 0 | 66 Gy | 1 |
| 3 | 93 weeks | 2 | Lip | T4N1M0 | 0 | 70,2 Gy | 2 |
| 4 | 412 weeks | 1 | Ethmoidal sinus | T4N2M0 | 1 | 60 Gy | 0 |
| 5 | 88 weeks | 1 | Cutaneous helix | T3N2M1 | 0 | 60 Gy | 1 |
| 6 | 90.86 weeks | 1 | Tongue | T4N0M0 | 1 | 60 Gy | 0 |
| 7 | 4.14 weeks | 3 | Floor of mouth | T4N2M0 | 0 | 70,4 Gy | 2 |
| 8 | 113.71 weeks | 1 | Tongue | T2N1M0 | 1 | 66 Gy | 1 |
| 9 | 24.14 weeks | 2 | Tongue | T2N0M0 | 1 | 0 Gy | 0 |
| 10 | 9.14 weeks | 3 | Tongue | T4N0M0 | 1 | 70 Gy | 1 |
| 11 | 2 weeks | 2 | Cutaneous helix | T2N2M0 | 0 | 70 Gy | 1 |
| 12 | 9.86 weeks | 2 | *Nasopharynx | T2N2M0 | 1 | 70 Gy | 1 |
| 13 | 49.29 weeks | 2 | Buccal mucosa | T4N2M0 | 0 | 70 Gy | 1 |
| 14 | 14.57 weeks | 2 | Mandible | T4N0M0 | 1 | 60 Gy | 0 |
| 15 | 62.43 weeks | 1 | Hypopharynx | T0N0M1 | 0 | 70 Gy | 1 |
| 16 | 10.71 weeks | 2 | *Nasopharynx | T0N0M1 | 0 | 0 Gy | 0 |
| 17 | 70.29 weeks | 1 | Tongue | T2N2bM0 | 0 | 70 Gy | 1 |
| 18 | 26 weeks | 1 | *Nasopharynx | T0N0M1 | 0 | 70 Gy | 1 |
| 19 | 11 weeks | 2 | Tongue | T3N1M0 | 1 | 61,8 Gy | 1 |
| 20 | 9.71 weeks | 3 | Tongue | T3N2bM0 | 1 | 70 Gy | 1 |
| 21 | 76 weeks | 2 | Oropharynx | T0N0M1 | 0 | 0 Gy | 0 |
| 22 | 27 weeks | 2 | Buccal mucosa | T3N0M0 | 1 | 67,5 Gy | 1 |
| 23 | 56 weeks | 1 | *Nasopharynx | T4N2cM0 | 0 | 70 Gy | 1 |
| 24 | 6.14 weeks | 2 | Tongue | T2N0M0 | 1 | 60 Gy | 0 |
| 25 | 9 weeks | 3 | Larynx | T0N0M1 | 1 | 0 Gy | 0 |
| 26 | 25 weeks | 1 | Tongue | T1N2aM0 ECE | 1 | 60 Gy | 1 |
| 27 | 5.86 weeks | 2 | Tongue | T1N2bM0 | 1 | 66 Gy | 1 |

*Nasopharynx cases only with keratinizing SCC pathology. ECOG PS- Eastern Cooperative Oncology Group Performance Scale; Cis- Cisplatin; Cet- Cetuximab.
